# N6-methyladenosine epitranscriptomic alterations and their potential implications for maternal–fetal immune tolerance in obstetric antiphospholipid syndrome

**DOI:** 10.3389/fimmu.2026.1936710

**Published:** 2026-10-29

**Authors:** Miguel A. Vázquez-Toledo, Carlos A. Guzman-Martin, Fausto Sánchez-Muñoz, Laura A. Matínez-Martínez, Yaneli Juárez-Vicuña

**Affiliations:** 1 Posgrado En Ciencias En Inmunología, Escuela Nacional de Ciencias Biológicas, Instituto Politécnico Nacional (ENCB-IPN), Mexico City, Mexico; 2 Doctorado en Ciencias Biológicas y de la Salud, Universidad Autónoma Metropolitana, Mexico City, Mexico; 3 Departamento de Fisiología, Instituto Nacional de Cardiología Ignacio Chávez, Mexico City, Mexico; 4 Departamento de Reumatología, Instituto Nacional de Cardiología Ignacio Chávez, Mexico City, Mexico; 5 Departamento de Inmunología, Instituto Nacional de Cardiología Ignacio Chávez, Mexico City, Mexico

**Keywords:** epitranscriptomics, m6A RNA methylation, maternal-fetal immune tolerance, METTL3, obstetric antiphospholipid syndrome, recurrent pregnancy loss, trophoblast dysfunction

## Abstract

Obstetric antiphospholipid syndrome (OAPS) is an autoimmune disease characterized by the persistent presence of antiphospholipid antibodies (aPL), such as anticardiolipin and anti-β2-glycoprotein I antibodies, in women experiencing obstetric complications. While vascular thrombotic events were considered the primary pathogenic mechanism in recent years, evidence indicates that the maternal-fetal interface in OAPS is altered by other underlying pathways, such as direct impairment of trophoblast invasion, dysregulation of immune cell activation, and platelet complement activation. These pathways act synergistically to compromise the immunological tolerance essential for fetal development. In parallel, recent research has highlighted the role of epigenetic modifications that maintain placental homeostasis and trophoblast proliferation. N6-methyladenosine (m6A) is the most abundant messenger RNA (mRNA) modification in mammals and plays a fundamental role in the maternal-fetal interface and the generation of immune tolerance. Specifically, the upregulation of m6A-modulating proteins is associated with recurrent pregnancy loss, defective placental artery remodeling, and HLA-G expression, a key factor in fetomaternal tolerance. Despite this knowledge, to date, no review has integrated m6A modification as a key mediator in the loss of immune tolerance in the context of adverse obstetric outcomes, including fetal growth restriction (FGR). In this review, we proposing a hypothetical model in which aPL exposure could induce alterations in the m6A methylation pattern that may affect the HLA-G/regulatory T cells (Treg)/uterine NK cells (uNK) axis potentially contributing to fetal loss, operating independently or in conjunction with prothrombotic mechanisms.

## 1 Introduction

The success of pregnancy depends on a dynamic balance between immunological tolerance to allogeneic fetal antigens and effective immune responses against pathogens. This delicate balance prevents the development of an immunosuppressive state and instead fosters a highly specialized process of immunological adaptation at the maternal-fetal interface. Here, signals from trophoblasts, decidual stroma, and immune system cells such as uterine NK cells (uNK), macrophages, and resident Tregs cells generate a tolerogenic microenvironment essential for the establishment and maintenance of the placenta (1, 2). However, disruptions in this balance can trigger adverse xobstetric events such as the development of preeclampsia, early pregnancy loss, and fetal growth restriction (3).

Among the main causes capable of disrupting this balance is OAPS, notable for being one of the leading causes of pregnancy-related complications. Traditionally, this autoimmune disease is associated with a prothrombotic microenvironment, induced by aPL when they are recognized by endothelial cells, monocytes, and platelets, leading to a hypercoagulable state that culminates in the formation of microthrombi in the intervillous space, compromising placental perfusion and fetal survival (4). Simultaneously, these antibodies promote the formation of neutrophil extracellular traps (NETs) and activation of the classical complement pathway, which recruits immune cells and generates a pro-inflammatory environment. In addition to these events, dysregulation of uNK cytotoxic function, imbalance in macrophage polarization, and decreased immunosuppressive capacity of Treg cells contribute to the loss of maternal-fetal immunological tolerance (5).

Despite advances in understanding the immunological mechanisms of OAPS, limitations remain in understanding other underlying mechanisms that regulate alterations in the immune response and homeostasis of the maternal-fetal interface (6). More recently, epitranscriptome has emerged as an important layer in the regulation of the immune response and fetal development. m6A represents the most abundant modification that occurs in mRNA in eukaryotic organisms, regulated by a set of methyltransferases (“writers”), demethylases (“erasers”), and reader proteins. These modify RNA stability, translation, and degradation. Furthermore, the m6A modification regulates fundamental cellular processes such as differentiation, activation, and response to different stimuli (7).

Recent studies demonstrate that the m6A modification plays a fundamental role in trophoblast activation and proliferation, decidualization (8, 9), placental implantation, and the remodeling of the immune microenvironment during pregnancy. Furthermore, the m6A modification participates in regulating innate and adaptive immune responses, specifically by regulating macrophage polarization and T-cell differentiation via the NF-κB and JAK/STAT pathways (10, 11). Specifically, the downregulation of the fat mass and obesity-associated protein (FTO) has been associated with a concomitant decrease in human Leukocyte Antigen G (HLA-G) expression in trophoblasts, a non-classical major histocompatibility complex (MHC) molecule that plays a central role in generating immunological tolerance by modulating the activation of uNK and Treg cells. Taken together, this evidence suggests that m6A dysregulation could influence the mechanisms that enable the generation of tolerance at the maternal-fetal interface (12). Studies demonstrate an association between alterations in m6A regulators and trophoblastic dysfunction and placental defects in some pathologies such as preeclampsia and recurrent miscarriages. However, the contribution of m6A modification in the context of antiphospholipid syndrome (APS) as a key regulator in the breakdown of maternal-fetal immune tolerance remains unexplored to date (13).

Given that OAPS is characterized by persistent inflammation, oxidative stress, and alterations in trophoblasts, evidence suggests that m6A modification acts as a potential regulator of these pathological processes. Based on this premise, we hypothesize in this review that dysfunction in m6A regulators could induce alterations in the functions of trophoblasts and decidual immune cells, promoting a pro-inflammatory microenvironment and contributing to fetal development and pregnancy loss (12, 14).

This review proposes a conceptual framework that integrates how the m6A epitranscriptomic modification contributes to the loss of immunological tolerance in the context of antiphospholipid syndrome (APS). First, it addresses the immunological mechanisms that maintain maternal-fetal tolerance, which are disrupted by the presence of antiphospholipid antibodies (aPL). Subsequently, it integrates evidence on the role of m6A during pregnancy, encompassing trophoblast function and HLA-G antigen-regulated tolerance generation, to link the m6A modification to the immunological mechanisms involved in APS. Finally, it proposes a possible model in which the m6A modification acts as a central axis in the immunological disruption at the maternal-fetal interface, highlighting its potential as a therapeutic target and as a biomarker.

A literature search was conducted across databases such as PubMed/MEDLINE, Scopus, Google Scholar, and Web of Science for articles published between January 2000 and May 1, 2026, with a focus on publications from the last five years. Search terms included combinations of “N6-methyladenosine” or “m6A” and “trophoblast,” “placenta,” “maternal-fetal tolerance,” “antiphospholipid syndrome,” “recurrent pregnancy loss,” “preeclampsia,” “HLA-G,” “regulatory T cells,” and “uterine natural killer cells.”

Reference lists of the retrieved articles were manually reviewed to identify relevant studies. Included articles comprised original research, mechanistic studies, and reviews in English addressing human tissues, cell lines, or animal models directly related to m6A regulation in reproductive or immunological contexts relevant to maternal-fetal tolerance. Conference abstracts, non-peer-reviewed preprints, and studies lacking direct relevance to m6A biology or maternal-fetal immunology were excluded. Given the lack of direct evidence linking m6A to obstetric antiphospholipid syndrome (OAPS), a portion of the cited literature was drawn from animal models, *in vitro* studies, and research related to other conditions such as preeclampsia and recurrent pregnancy loss (**Figure 1**).

**Figure 1:**
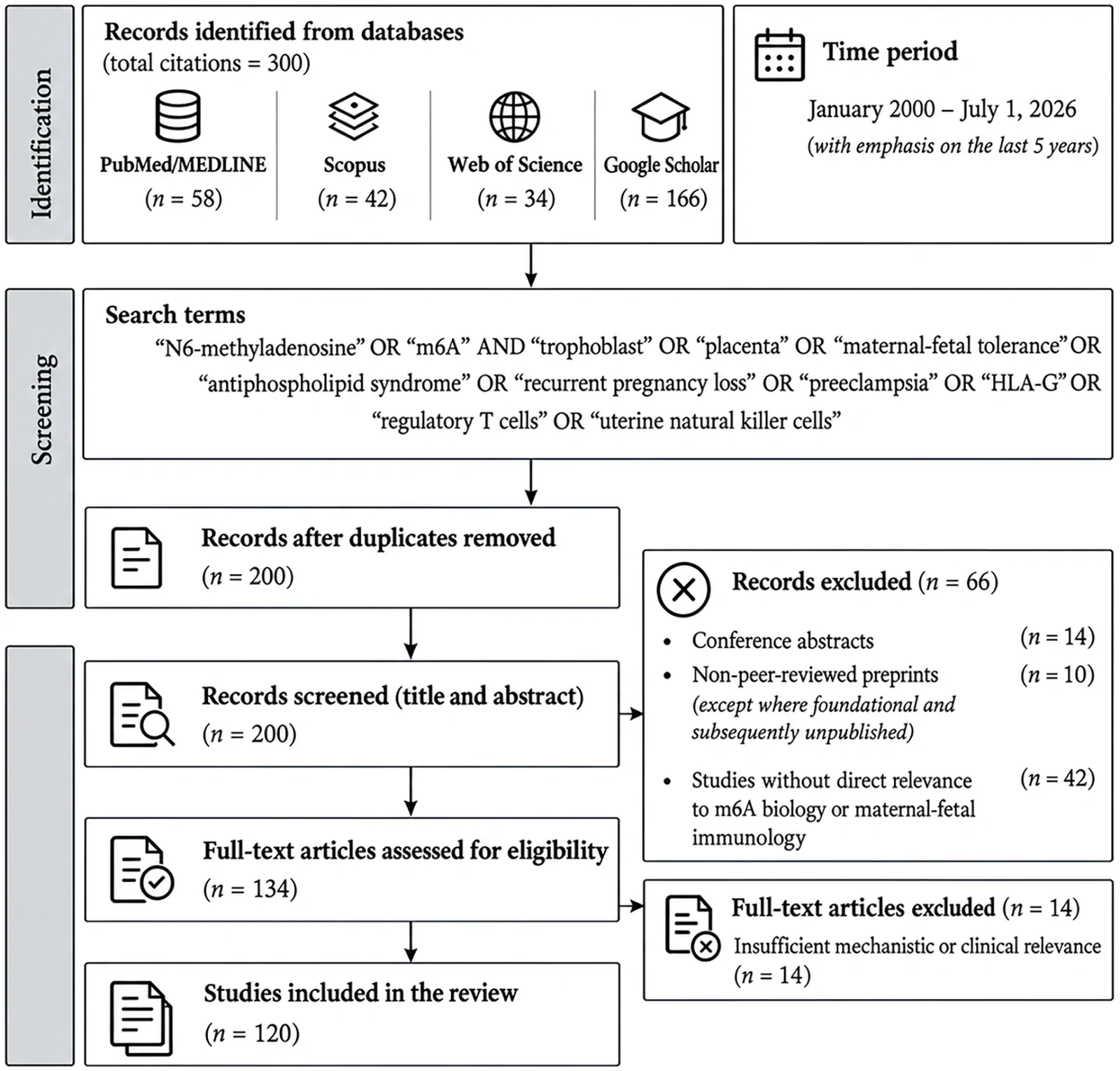
Literature search and study selection process. Narrative literature search conducted in PubMed/MEDLINE, Scopus, Web of Science, and Google Scholar (January 2000–July 1, 2026), followed by duplicate removal and screening according to predefined eligibility criteria. A total of 300 records were identified and 120 studies were included in the review, with relevant evidence from related pregnancy disorders considered because of limited direct OAPS data. Created using SciDraw (sci-draw.com).

## 2 Obstetric APS: pathogenesis beyond thrombosis

### 2.1 Antiphospholipid antibodies: targets, isotypes, and pathogenic heterogeneity

aPLs are part of a heterogeneous family of immunoglobulins directed against proteins with high affinity for phospholipids, rather than against the phospholipids themselves. Among the main molecules targeted by aPLs are β2 glycoprotein I (β2GLPI), cardiolipin, and phospholipid-protein complexes exposed on the cell surface during stress, apoptosis, or activation (15). β2GLPI is a plasma glycoprotein that, under normal physiological conditions, circulates in a closed conformation. However, it adopts an open structural conformation upon binding to negatively charged phospholipids such as phosphatidylserine, which is externalized during apoptosis or cell activation. This conformational change exposes epitopes that remain hidden in their domain. These epitopes are highly immunogenic and closely related to obstetric morbidity (16).

The relevance of different aPL subtypes in the pathogenesis of OAPS is significantly different between the thrombotic and obstetric manifestations of the syndrome, suggesting the existence of divergent pathophysiological mechanisms (17). These differences lie in their isotypic and subclass distribution, which influence their functions, particularly IgG1 and IgG3 antibodies. These antibodies are characterized by their ability to interact with Fcγ receptors, activating the classical complement pathway, which allows the release of chemoattractant, amplifying the inflammatory process. In contrast, IgM antibodies tend to be more polyreactive and have lower affinity, while IgA aPL participate in Fc domain-independent mechanisms that are not yet fully understood (18). These differences underscore that aPL have different functions that depend largely on their isotype and affinity, which confers their variable pathogenic capacity.

### 2.2 Complement activation and neutrophil extracellular traps

Among the main mechanisms unrelated to thrombotic outcomes in OAPS is complement activation, which has been revealed as one of the main pathological mechanisms of the syndrome. Studies in murine models of aPL-induced pregnancy loss demonstrated that complement activation is not a secondary event, but a fundamental process in fetal loss. In C3(-/-) knockout mice or mice treated with complement inhibitors, a protective effect was shown, allowing them to reach term despite having similar circulating aPL levels to wild-type mice (19).

C5a has been shown to be a key mediator, an anaphylatoxin that enables the recruitment of neutrophils, macrophages, and decidual cells via the C5a receptor (C5aR). This accumulation of neutrophils promotes the generation of a pro-inflammatory microenvironment characterized by the release of reactive oxygen species, proteases, and tissue factors (TF), hindering trophoblastic cell invasion and disrupting the maternal-fetal interface (20).

The culminating product is the formation of the membrane attack complex (MAC), which amplifies damage to trophoblastic and endothelial cells by depositing directly onto plasma membranes. This induces apoptosis and the externalization of phosphatidylserine, creating additional binding sites for aPL and thus a self-perpetuating cycle of complement activation and inflammation (21). Furthermore, deposits of complement waste products such as C3b, C4D, and MAC have been consistently found in placental biopsies from pregnant patients with OAPS, further strengthening their role in this syndrome and providing a distinctive marker of placental injury separate from the thrombotic process (4) (**Figure 2D**).

**Figure 2:**
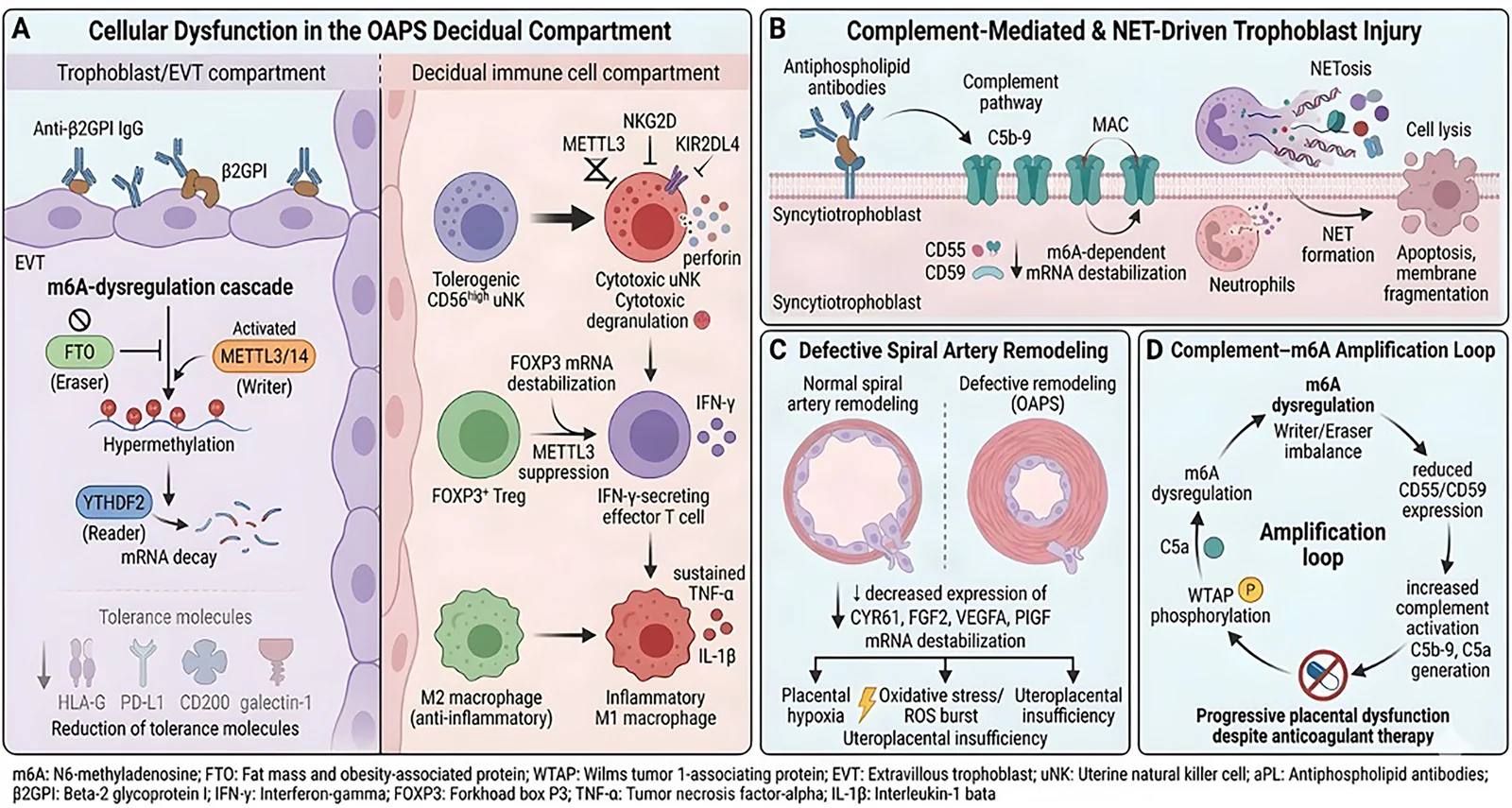
Altered maternal-fetal immune tolerance in obstetric APS. **(A)** In the model proposed here, binding of aPL/anti-β2GPI to the trophoblast is hypothesized to induce m6A dysregulation, which may promote degradation of tolerogenic mRNA molecules (HLA-G, PD-L1, CD200, galectin-1) and the transition to an immunogenic phenotype. This change is associated with cytotoxic activation of uNK cells, destabilization of Tregs (loss of FOXP3), and polarization of macrophages toward a pro-inflammatory M1 phenotype. **(B)** Complement activation and NET formation induce direct trophoblast damage, exacerbated by the decrease in regulators such as CD55 and CD59. **(C)** Altered stability of pro-angiogenic transcripts compromises trophoblastic invasion and spiral artery remodeling, favoring hypoxia and placental insufficiency. **(D)** The interaction between complement activation and m6A dysregulation establishes a feedback loop that perpetuates placental dysfunction.

NETs are reticular structures of decondensed chromatin, composed of histones, elastases, myeloperoxidase, and proteins with antimicrobial activity, released by neutrophils after activation by aPL through a process known as NETosis (22, 23). In the case of OAPS, studies reveal that NETs released by aPL recognition expose β2GPI in their chromatin scaffold in an open conformation that allows for greater binding of autoantibodies, directly damaging the syncytiotrophoblast membranes in a contact-dependent manner. Additionally, transcriptomic analyses of single cells from the decidua of women with OAPS have demonstrated a marker with an enrichment of genes associated with neutrophil activation programs, such as citrullinated histone H3 and peptidylarginine deiminase 4 (PAD4), in the trophoblast invasion zones (20). This relationship between NETs in the decidual membrane and defective EVT invasion implies a strong influence of NETosis on placental failure, operating independently of intravascular thrombosis (**Figure 2B**) (24).

### 2.3 Disruption of the immune microenvironment at the maternal-fetal interface

Antibody-mediated complement activation, the formation of extracellular traps, and direct trophoblast injury act synergistically to alter the immunological microenvironment of the maternal-fetal interface, shifting the tolerogenic environment of the decidua to a pro-inflammatory state that affects fetal development (25) (**Figure 2C**). Transcriptomic analyses at the single-cell level have revealed that these alterations are not uniform but rather reflect programming in multiple cell populations of the immune system, which contribute with specific mechanisms but converge toward the loss of immunological tolerance (26).

Decidual resident macrophages undergo a change in their phenotype, polarizing from an M2 (anti-inflammatory) phenotype to an M1 (pro-inflammatory) state, characterized by the release of pro-inflammatory cytokines including TNF-α, IL-1β, IL-6, and inducible nitric oxide synthase (27). This change in macrophage phenotype alters regular mechanisms essential for the invasion of extravillous trophoblasts and the maturation of NK cells. Furthermore, uNK cells increase the expression of activating receptors such as NKG2D and NKp46 and exhibit reduced inhibitory signaling, which promotes aberrant cytotoxic responses against trophoblastic cells (**Figure 2A**) (28).

In addition, the reduced expression of HLA-G in trophoblasts eliminates a key signal for inhibiting the exacerbated immune response, reinforcing a feedback loop that amplifies cellular damage (29). Simultaneously, the frequency and function of Tregs decrease, exhibiting reduced FOXP3 stability, decreased IL-10 production, and reduced expression of the IL-2 receptor, which is fundamental for controlling the proliferation of effector T cells, thus weakening central mechanisms of immunological tolerance (30).

Finally, alterations in the decidual immunological microenvironment allow B cells to polarize into antibody-producing plasma cells in the local tissue, suggesting that local aPL production can amplify the inflammatory process directly at the maternal-fetal interface (31). The convergence of macrophage polarization to a pro-inflammatory phenotype, increased cytotoxic activity of uNK cells, decreased Tregs, and local antibody production generate a microenvironment conducive to loss of tolerance, which could explain the limited success of current therapies focused on modulating the procoagulant microenvironment (32). In this context, epitranscriptional modifications emerge as a key initial regulator because many mechanisms regulated by m6A have the ability to orchestrate post-transcriptional programs that regulate trophoblast functions, immune cell phenotype, and tolerogenic pathways such as HLA-G molecule expression, indicating that m6A modification may contribute to the regulation of in immune balance during pregnancy (10, 33).

## 3 The m6A epitranscriptome: writers, erasers, and readers

### 3.1 Biology of m6A: structure, prevalence, and dynamic regulation

Reversible chemical modifications of mRNA provide an extra layer, an extra layer of regulation independent of changes in the DNA sequence (34). There are approximately 170 known RNA modifications to date, with the addition of a methyl group to the nitrogen atom at position 6 of the adenosine ring standing out as the most abundant and having the greatest functional impact (35). In mammals, m6A represents the most frequent modification occurring in polyadenylated mRNA and long non-coding RNAs, estimated to be found in approximately 0.1% to 0.6% of all adenosine residues carrying the modification, with an average of 3 to 5 m6A sites per individual transcript. It exerts different functions such as cell differentiation, stress adaptation, and immunological function (36).

m6A methylation follows a highly conserved pattern throughout the transcriptome. Modification sites are primarily located within a DRACH consensus sequence motif, enriched in specific mRNA regions, including areas near stop codons, long internal exons, and the 3′ untranslated region (3′UTR), with marked accumulation at the junction of the last exon with the 3′UTR. This spatial distribution is particularly important, as the regulatory outcome of m6A depends on its location within the transcript (7, 37). Methylation sites located in 3′-UTR regions primarily regulate mRNA stability and translation efficiency, while those found in coding and proximal regions modulate translation, protein co-translation, and alternative splicing. Therefore, the m6A modification can impact the functionality of transcripts not only through their expression but also through their distribution within the RNA structure (38).

The main difference of m6A is its dynamic and reversible nature, which distinguishes it from other epigenetic modifications such as RNA methylation and histone modification. While DNA changes typically occur over long periods, m6A modifications are deposited and cleared within hours in response to stimuli or cellular stress, allowing for rapid and reversible transcriptomic reprogramming. This aspect has a significant impact in dynamic biological contexts such as trophoblast differentiation, immune response, the maternal-fetal interface, and stromal remodeling, which require transcriptional changes within specific time windows (39, 40). This capacity for rapid, sensitive, and finely tuned regulation in response to different stimuli suggests that m6A may represent an important regulatory layer, as alterations in m6A homeostasis could potentially contribute to pathological processes relevant to OAPS, although this specific relationship remains to be established.

### 3.2 Writers: METTL3, METTL14, WTAP, and the methyltransferase complex

The deposition of m6A onto mRNA is catalyzed by enzyme complexes with multicomponent nuclear methyltransferase activity whose core structure is highly conserved in eukaryotes (41). At the center of this complex is methyltransferase type 3 (METTL3), an enzyme that uses S-adenosylmethionine as a cofactor, which acts as a methyl group donor at the N6 position of adenosine within DRACH motifs. For METTL3 to perform its function, it must be in the form of a heterodimer with methyltransferase type 14 (METTL14), a catalytically inactive enzyme that stabilizes the functional conformation of METTL3. Together, they act as the main RNA-binding scaffold. This heterodimer is coordinated by Wilms tumor-associated protein 1 (WTAP), which defines the location of the complex and couples m6A deposition with co-transcriptional RNA processing events (42).

On the other hand, several accessory molecules increase the substrate specificity and spatial localization of m6A deposits. Among the main ones are the RNA-binding motif 15 (VRMA) protein and its homologous gene RBM15B, which regulates the recruitment of the methyltransferase complex to transcripts with specific sequences through the recognition of uracil-rich (U) regions, primarily in RNA and lncRNA. VIRMA, also known as KIAA1429, directs methylation to the 3’ UTR region and regions adjacent to the stop codon by interacting with the cleavage and polyadenylation complexes, thus promoting methylation in these characteristic regions of these transcripts (43).

Finally, ZC3H13 stabilizes the nuclear localization of the complex and is involved in mRNA export, ensuring that the m6A modification is carried out properly before processing in the cytoplasm. Together, these components form a highly coordinated system that modulates sequence recognition, reorganization, and spatial regulation of RNA methylation. Beyond their structural characteristics and conformation, these protein complexes acquire great relevance for carrying out specific biological functions, particularly at the maternal-fetal interface (44).

An important factor in the reproductive immune response that has emerged in recent years is METTL3, which directly influences trophoblast function and the maternal-fetal interface. During early gestation, cytotrophoblasts express high levels of METTL3, coinciding with the period of maximum extravillous trophoblast invasion, a process that decreases as placentation progresses (45). Studies have shown that the loss of METTL3 in trophoblastic lineages results in defective placentation due to impaired invasion, reduced uteroplacental perfusion, and consequent fetal growth restriction (46).

The proposed mechanism is that METTL3 deficiency promotes the destabilization of transcripts encoding pro-invasive factors such as FGF2, VEGFA, and MYLK, compromising cytoskeleton remodeling, extracellular matrix degradation, and angiogenic signaling (47). It´s worth noting that METTL3 deficiency has been demonstrated in human trophoblast stem cell and murine models, and that aPL-induced trophoblastic dysfunction has been independently shown in obstetric APS, characterized by defective arterial remodeling and fetal growth restriction (48, 49). Whether both processes converge via a common m6A-dependent pathway in obstetric APS—as proposed in the integrative model in Section 5—has not yet been experimentally verified.

### 3.3 Erasers: FTO and ALKBH5 as dynamic demethylation regulators

The reversibility of the m6A modification is regulated by enzymes with demethylase activity that catalyze the oxidative removal of the methyl group from the modified adenosine residues, allowing for rapid and sensitive reprogramming in response to different stimuli or cellular stress. The two main enzymes responsible for regulating this process are FTO and the 5 AlkB homolog (ALKBH5), both of which belong to the superfamily of iron-dependent (Fe^2+^) and α-ketoglutarate-dependent dioxygenases (50). These enzymes require molecular oxygen (O_2_) as a cofactor to carry out their enzymatic activity, making them sensitive to changes in cellular metabolism and oxidative stress, conditions related to the pathophysiology of OAPS.

The first demethylase characterized was FTO, identified as a key regulator of energy metabolism and adipogenesis before its ability to remove methyl groups from RNA was discovered (51). In this context of maternal-fetal interface development, FTO emerges as a key regulator through its ability to influence the stability of HLA-G mRNA in trophoblasts by maintaining a hypomethylated state that allows interaction with reader proteins such as IGF2BP1. This interaction preserves HLA-G expression on the EVT membrane, thus preventing uNK cell-mediated cytotoxicity. However, when FTO activation is reduced, either by proinflammatory cytokine signaling or oxidative stress, leading to increased methylation of HLA-G transcripts, accelerated mRNA degradation by degradation-associated readers such as YTHDF2 is promoted (52). Its alteration by aPL-mediated inflammatory signals is detailed in Section 6, providing a plausible, though not yet directly demonstrated, molecular explanation for the decrease in HLA-G expression consistently observed in biopsies from patients with OAPS.

In contrast, ALKBH5 differs from FTO primarily in its subcellular localization, as it is preferentially found in the nucleus rather than the cytoplasm, and its preferred substrates are transcripts that regulate the cell cycle, RNA processing, and cell invasion (53). In trophoblasts, ALKBH5 regulates the stability of CYR61 mRNA, which encodes proteins essential for cell adhesion, cytoskeleton reorganization, and cell migration. In homeostasis, the balance between methylation and demethylation mechanisms maintains optimal CYR61 expression and allows for trophoblast invasion. However, the increase in ALKBH5 expression shifts the equilibrium toward a hypomethylated state, causing CYR61 transcripts to lose stability, thus reducing the expression of proteins necessary for trophoblast invasion (22). It is worth noting that the decrease in trophoblast invasion mediated by aPL and ALKBH5 may be occurring through alterations in metalloproteinase activity, modifications in the cytoskeleton, and altered interaction between EVT and the decidua, supporting the idea that FTO and ALKBH5 act as regulators of immunological tolerance (54).

### 3.4 Readers: the YTHDF family, IGF2BPs, and post-transcriptional fate determination

The effects of the m6A modification are determined by reader proteins that recognize and decode methylated transcripts, translating the signal into post-translational changes that directly affect mRNA stability, translation, and intracellular localization. These proteins comprise two main families: the first contains the YTH domain, and the second family consists of IGF2BP mRNA-binding proteins, representing the main cytoplasmic readers and exerting largely opposing regulatory effects (55).

The THY family of proteins recognizes the m6A modification through their conserved domain, which has high specificity for recognizing methylated adenosine. The main function of YTHDF2 is to promote mRNA degradation by recruiting the CCR4-NOT complex, while YTHDF1 facilitates translation by promoting ribosome binding. YTHDF3 acts as a modulator of YTHDF1 and YTHDF2 activity, enhancing the translation of methylated transcripts in cooperation with YTHDF1, while in cooperation with YTHDF2 it facilitates their degradation (56).

In the nucleus, YTHDF1 regulates position-dependent alternative splicing; the position of m6A allows the recruitment of SRSF3 to add exons or SRSF7 to omit exon inclusion. This function is highly relevant for the generation of HLA-G isoforms, which are distinguished from each other by their membrane binding, secretion, and immunological activity at the maternal-fetal interface (57). Conversely, IGF2BP proteins stabilize methylated transcripts and promote translation by sequestering them within cytoplasmic ribonucleoprotein complexes. Unlike YTHDF2, IGF2BP proteins recognize methylated sites through their K homology (KH) domain and subsequently recruit them into cytoplasmic stress granules and P bodies, where they are protected from degradation or stored until later translation. This stabilizing function is crucial under conditions of cellular stress and inflammation, where the preservation of key transcripts becomes essential (58).

In the context of obstetric complications, reduced expression of IGF2BP1 and IGF2BP2 in chorionic villus tissue has been associated with downregulation of HLA-G, suggesting that this mechanism may be contributing to the loss of tolerance. In the inflammatory microenvironment, the activity of proinflammatory cytokines causes an imbalance between stabilizing readers such as IGF2BP and destabilizing readers such as YTHDF2, altering the degradation of tolerance-associated transcripts. This imbalance provides an overview in which the regulation of m6A readers integrates fundamental regulatory signals in the regulation of gene expression programs crucial to trophoblast functioning and maintenance of immune homeostasis (59).

### 3.5 Role of m6A in innate and adaptive immune cells

The regulatory role of the m6A modification is not limited to trophoblast biology; it affects a wide variety of immune cell populations relevant to maintaining maternal-fetal homeostasis, positioning m6A as a central regulator of the decidual microenvironment (60). In innate immune cells, the m6A modification regulates pattern recognition receptor (PRR) signaling, cytokine production, and inflammasome activation—functions directly related to macrophage polarization and the cytotoxic activation of uNK cells characteristic of the placental decidua (61).

In macrophages, the stability of transcripts encoding anti-inflammatory mediators is regulated by METTL3, such as IL-10 and suppressor of cytokine signaling 1 (SOCS1). Conversely, the degradation of anti-inflammatory cytokine mRNA is directed by YTHDF2, allowing macrophages to polarize to their pro-inflammatory phenotype (M1) in response to inflammatory signals such as lipopolysaccharide (LPS) (62). In contrast, the loss of METTL14 in macrophages directly affects their ability to polarize to an anti-inflammatory phenotype (M2) in response to IL-4, indicating that macrophage plasticity is largely determined by the activity of m6A regulators (63). In the decidual microenvironment, inflammation triggered by aPL may be related to alterations in METTL3 activity in decidual macrophages, promoting sustained M1 polarization by suppressing anti-inflammatory transcripts, even in the absence of the initial aPL stimulus (64).

In NK cells, m6A modifications regulate the expression of activating and inhibitory (NKG2A) and activating (NKG2D) receptors through transcriptional and post-transcriptional mechanisms. METTL3 regulates the expression of transcripts encoding activating receptors such as NKG2D, the main receptor by which NK cells recognize transformed cells, thereby modulating their activation (65). In the decidual context, NK cells play a fundamental role because the imbalance between KIR/NKG2A-regulated inhibition and NKG2D-mediated activation determines the ability of uNK cells to attack or tolerate antigens on trophoblastic cells. Disruption of this balance is determined by m6A and inflammatory signals associated with antibody activity, which allow uNK cells to generate cytotoxic activity against trophoblasts in the absence of inhibitory signals such as HLA-G expression (66).

In Treg cells, m6A modifications lead to the stabilization of the FOXP3 master transcription factor mRNA, a characteristic of the Treg lineage and its suppressor function. METTL3 promotes the stability of FOXP3 transcripts through IGF2BP proteins, ensuring sustained expression so that they can exert their immunosuppressive activity. However, in inflammation induced by complement activation and the activity of proinflammatory cytokines associated with aPL signaling, METTL3 expression is suppressed in Tregs via the NF-κB signaling pathway, reducing m6A methylation levels in FOXP3 transcripts, thus facilitating the transition of Treg cells to an effector phenotype (67, 68).

In B cells, m6A modification regulates fundamental processes in humoral immunity, such as isotype switching, somatic hypermutation, and differentiation into antibody-producing plasma cells, by controlling AID mRNA. METTL14-mediated alterations in AID can promote the expansion of autoreactive clones of anti-β2GPI IgG-producing B cells (69). Taken together, these mechanisms support the role of m6A as an initial regulator of immune balance, although they have not been directly linked in the context of OAPS, which links the alteration in the immune and adaptive response with the persistent production of autoantibodies.

## 4 m6A at the maternal-fetal interface: evidence from placental biology

### 4.1 METTL3-dependent m6A writing controls trophoblast invasion and spiral artery remodeling

The m6A modification plays a fundamental role during placental development, coordinating a complex network of cellular and molecular interactions that regulate trophoblast differentiation, proliferation, and invasion, as well as vascular remodeling and the remodeling of the decidual immune microenvironment. Recent studies suggest that alterations in the methylation profile can trigger pathological processes in the absence of direct immunological stimuli (10).

The importance of m6A in placental maintenance lies in the coordination of m6A regulatory enzymes. METTL3 is an enzyme highly expressed during the first trimester of pregnancy, especially in progenitor cytotrophoblasts located in the villi and in the initial phase of their differentiation into invasive endothelial trophoblasts. However, as they migrate and acquire a terminal phenotype, METTL3 expression decreases, suggesting that they have a specific role at each stage of development that allows for functional maturation (70).

The mechanisms by which METTL3 regulates trophoblast invasiveness involve MYLK, a molecule that mobilizes the cytoskeleton; FGF2, which activates the PI3K-AKT pathway to promote cell survival; matrix metalloproteinases 14 (MMP-14), which enables the degradation of the extracellular matrix; and VEGFA, an angiogenic signal. Loss of METTL3 leads to a global disruption of these mechanisms of trophoblast migration, invasion, and maturation (71).

Furthermore, the consequence of METTL3 depletion directly affects the establishment of low resistance uteroplacental circulation. The m6A modification of FGF2 mRNA allows its secretion into the decidual stroma, where it acts as a paracrine signal, enabling endothelial trophoblast invasion and smooth muscle dedifferentiation (45). In METTL3 (-/-) mouse trophoblasts, loss resulted in contractile spiral arteries, leading to hypoxia, placental hyperfusion, and maternal hypertension. Complementarily, the decrease in PlGF causes alteration in angiogenesis characterized by a reduced PlGF/sFlt-1 ratio, a phenomenon shared by preeclampsia (72).

### 4.2 ALKBH5–CYR61 and FTO–HLA-G axes: regulation of invasion and immunological tolerance

ALKBH5 and FTO are key enzymes that link m6A modification to key trophoblast functions. ALKBH5 has been associated with pregnancy loss through the regulation of CYR61 mRNA, a protein involved in cell adhesion and angiogenesis. However, overexpression of ALKBH5 leads to the deletion of m6A in the 3′UTR region of CYR61, interfering with the binding of IGF2BP and stabilizing proteins, causing their degradation. Furthermore, it compromises the integrin-FAK interaction, reducing the invasive capacity of the trophoblast (22).

ALKBH5 regulation is controlled by HIF-1α activation in response to placental hypoxia and NF-κB activation induced by proinflammatory cytokines. This generates a feedback loop in which vascular dysfunction increases hypoxia, increases ALKBH5 expression and perpetuating the invasive defect (73). Moreover, FTO regulates the hypomethylation of HLA-G mRNA to maintain its stabilization and expression in trophoblasts. Pro-inflammatory signals and oxidative stress lead to a reduction in FTO activity, causing hypermethylation of these transcripts and their subsequent degradation mediated by YTHDF2, thus decreasing HLA-G expression (**Figure 3**). This reduction allows for greater cytotoxic activity of decidual NK cells against the trophoblast. Furthermore, recent studies demonstrate that IgG derived from patients with APS directly reduces FTO expression in trophoblasts (74). Finally, the loss of FTO induces the coordinated degradation of other molecules that allow the generation of immunological tolerance such as HLA-E, PD-L1 and galectin-1, proving that trophoblasts can be recognized as “stranger” and mount immune responses (52).

**Figure 3:**
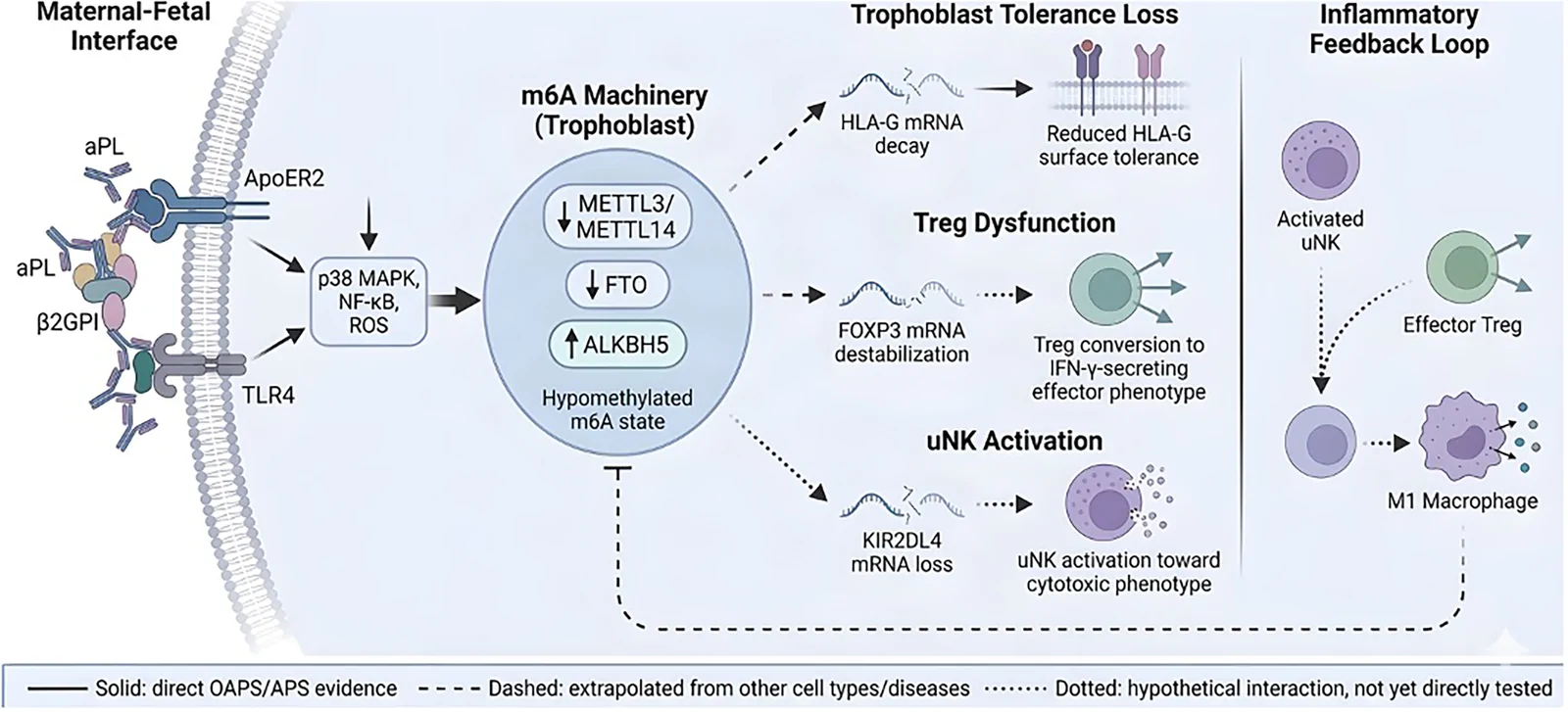
Proposed model linking aPL to maternal–fetal immune tolerance breakdown through m6A dysregulation in OAPS. Anti-β2GPI immune complexes activate ApoER2/TLR4 signaling in trophoblasts, triggering p38 MAPK, NF-κB, and oxidative stress pathways that disrupt the m6A machinery (↓METTL3/METTL14, ↓FTO, ↑ALKBH5). Altered m6A homeostasis promotes destabilization of HLA-G, FOXP3, and KIR2DL4 transcripts, resulting in impaired trophoblast-mediated tolerance, loss of Treg stability, and activation of cytotoxic uNK cells. These changes favor M1 macrophage polarization and inflammatory cytokine production, establishing a self-amplifying feedback loop that perpetuates immune dysregulation and contributes to maternal–fetal immune tolerance collapse in OAPS.

### 4.3 Regulation of decidual immune cells by m6A and amplification of tolerance failure

m6A regulates the polarization, activation, and effector functions of cell populations such as NK cells, macrophages, and T cells. In uNK cells, m6A methylation favors a tolerogenic proangiogenic phenotype characterized by the release of angiogenic mediators such as VEGFA and PlGF (75). However, in the antibody-induced proinflammatory microenvironment, MTLL3 expression decreases, reducing the production of these proangiogenic factors and leading to a transition to a cytotoxic phenotype characterized by the secretion of perforins and granzymes (76). Similarly, alterations in m6A modification cause a decrease in the expression of the inhibitory receptor KIR2DL4, which, together with the decrease in HLA-G, results in a failure to inhibit the cytotoxicity of NK cells against EVT (48).

In decidual macrophages, m6A regulates M1 and M2 phenotype polarization by controlling SOCS1 and SOCS3, and negative regulators of JAK-STAT. Suppression of METTL14 in response to inflammatory stimuli decreases the stability of these transcripts, allowing STAT1 activation and polarization to an M1 phenotype (63). Taken together, these mechanisms demonstrate that m6A dysregulation not only affects trophoblast function but also regulates multiple cellular programs that integrate key functions in trophoblast development and immunological tolerance.

## 5 m6A dysregulation as a candidate mechanism contributing to immune tolerance loss in obstetric APS: a proposed integrative model

### 5.1 Three convergent mechanisms through which aPL induce m6A dysregulation in trophoblasts

Demonstrating that aPL induces dysregulation of m6A modification in trophoblasts requires a mechanistic context that extends beyond aPL-mediated injury and the alteration of m6A regulators to impaired placental function. In this context, the central question is whether the binding of anti-β2GPI antibodies can activate intracellular signaling pathways capable of modifying the expression, activity, and substrate specificity of the enzymes that regulate m6A. Current evidence supports this possibility, particularly through anti-β2GPI antibody-induced signaling on the trophoblast surface.

Immune complexes (anti-β2GPI/β2GPI) associated with trophoblast membranes are recognized by Toll-like receptor 4 (TLR-4) and ApoER2, leading to the activation of the p38 MAPK and NFκB pathways—a phenomenon demonstrated in patients with thrombotic APS; however, their specific downstream effect on m6A writers and erasers has so far only been shown in a non-placental cell model of hypoxia/reoxygenation (21, 49). It has been shown that p38 MAPK promotes the post-translational destabilization of METTL14 by phosphorylating its RNA-binding domain at serine residues, thereby reducing its affinity for target transcripts and the stability of METTL3; this has been demonstrated in LPS-stimulated macrophages and IL-6-stimulated periodontal ligament cells (77, 78).

In parallel, NF-κB exerts broader transcriptional effects by suppressing METTL3 expression through conformational modification of chromatin associated with the METTL3 promoter, allowing the recruitment of the histone deacetylase HDAC1 to generate a closed chromatin conformation of the METTL3 locus, while simultaneously inducing ALKBH5 transcription, which shifts the epitranscriptome to a hypomethylated state (77, 78).

The third regulatory mechanism through which aPLs might contribute to m6A dysregulation involves mitochondrial dysfunction and oxidative stress. Receptors such as annexin A2 and ApoER2 trigger NADPH oxidase activation and phosphorylation of the p47phox subunit, thereby increasing the production of reactive oxygen species (ROS) in patients with APS. These ROS induce mitochondrial Ca^2+^ overload, causing membrane potential depolarization and pore opening, which promote disruption of the electron transport chain and ROS overproduction (79). This oxidizing environment leads to the oxidation of the Fe^2+^ cofactor to Fe^3+^ of FTO and ALKBH5, inactivating the catalytic activity of both demethylases and increasing the methylation of specific transcripts such as HLA-G mRNA, whose stability is associated with hypomethylation under physiological conditions. Evaluated in other cell populations and not directly in trophoblasts. Together, these mechanisms, each demonstrated separately in isolated experimental systems, raise the possibility of a convergent pathway linking aPL exposure to m6A dysregulation and loss of maternal-fetal tolerance; a direct causal link has not yet been established in OAPS and remains to be tested (80–82).

### 5.2 m6A-driven HLA-G loss and the collapse of the trophoblast tolerance shield

The mechanism linking aPL-induced m6A dysregulation and impaired tolerance is through HLA-G regulation. HLA-G is a molecule highly expressed in EVTs that restricts maternal immune activation against fetal antigens. The stability of its transcripts depends on FTO-regulated hypomethylation, the degradation of which is mediated by YTHDF2, which determines HLA-G expression. Disruption of this balance by changes in m6A regulatory enzymes induced by the presence of aPL could promote changes in m6A density in HLA-G mRNA, favoring its degradation and consequently reducing its expression, a phenomenon observed in the context of idiopathic miscarriage (52).

The consequences of this reduction in HLA-G expression are diverse and have been described primarily in the context of recurrent implantation failure. In homeostasis, HLA-G interacts with the inhibitory receptor ILT2 on decidual uNK cells, transmitting signals that regulate the recruitment of the ITIM-dependent SHP-1 phosphatase, repressing the release of granzymes and cytotoxic agents against trophoblasts (83). However, when its expression decreases following FTO suppression induced by the presence of aPL, the inhibitory signal provided by ILT2 is insufficient to counteract the activating signals promoted by the interaction of NKG2D with ligands overexpressed in aPL-damaged trophoblasts, causing the transition of uNK cells to a cytotoxic phenotype against EVT (29).

Furthermore, the loss of HLA-G also affects other immunological mechanisms mediated by other cell populations. Activation of ILT4 in decidual macrophages normally induces IL-10 production, characteristic of the M2 anti-inflammatory phenotype (84). On the other hand, the interaction of HLA-G and ILT2 in decidual dendritic cells inhibits the expression of costimulatory molecules (CD80/CD86), thus preventing the activation of T cells against fetal antigens (85). The binding of HLA-G to KIR2DL4 on uNK cells promotes a tolerogenic phenotype characterized by the production of IL-10, VEGFA, and IFN-γ. Therefore, the inhibition of HLA-G expression mediated by m6A not only involves the elimination of inhibitory signals but also compromises a complex signaling network (86).

### 5.3 m6A dysregulation and Treg destabilization in the OAPS decidua

APS is characterized by a reduced frequency of decidual FOXP3+ Treg cells and suppressive Treg dysfunction, attributed to the pro-inflammatory microenvironment induced by complement activation triggered by the presence of aPL and cells of the innate immune system. While this phenomenon is attributed to complement activity and pro-inflammatory cytokines, m6A dysregulation may offer an additional, hypothetical explanation for the reduced Treg cell frequency, one that has not yet been directly tested in OAPS and is currently supported only by evidence from other autoimmune conditions (87).

As mentioned in section 3.5, FOXP3 mRNA stability depends on m6A, which promotes the stabilization of the IGF2BP2 and IGF2BP3 transcripts, maintaining FOXP3 expression necessary for suppressive activity. In the context of autoimmune diseases such as systemic lupus erythematosus (SLE), chronic NF-κB activation—induced by antibodies or via the C5a receptor (RC5a) in Treg cells suppresses METTL3 expression, reducing m6A in FOXP3 mRNA. Consequently, Treg cells polarize to an effector phenotype induced by the production of IFN-γ and the transcription factor T-bet and the loss of IL-10 secretion, contributing to an inflammatory state similar to that reported in patients with head and neck cancer (88).

This mechanism is of vital importance because it can self-amplify and is potentially reversible. The IFN-γ produced by Tregs can further suppress METTL3 expression in adjacent cells through JAK1 and STAT1 signaling, perpetuating FOXP3 instability within the decidual microenvironment (89). However, since this process acts at the post-transcriptional level, the restoration of MTLL3 and m6A homeostasis could theoretically recover FOXP3 expression before irreversible epigenetic modifications of the FOXP3 locus-similar to what has been observed in patients undergoing tumor immunotherapy (90, 91).

### 5.4 Complement activation and its intersection with the m6A pathway

One of the central mechanisms of non-thrombotic pathology in placental abruption associated with fetal loss is the complement system, and its interaction with m6A can generate a feedback loop that bidirectionally amplifies the alteration of the maternal-fetal microenvironment (92). Additionally, the products released by complement activation can modify m6A regulators in trophoblasts and decidual immune cells. Eventually, the alteration of m6A can reduce complement regulatory proteins, allowing for greater activation and tissue damage (93).

A potential link between the complement system and m6A has been observed in the context of placental dysfunction and C5a-mediated preeclampsia. The binding of C5a to its receptor (C5aR) in trophoblasts activates signaling pathways dependent on Gαi-coupled phospholipase C, the induction of protein kinase C (PKC), and the subsequent phosphorylation of p38 MAPK (94). These pathways modify the regulation of m6A through interaction with methyltransferases like those described in section 4.1. Studies demonstrate that PKC activation can phosphorylate WTAP, the subunit responsible for scaffolding the methyltransferase complex, affecting its interaction with METTL3 and METTL14, thus impacting the nuclear localization of the entire complement complex (91, 95).

The bidirectional interaction between the complement system and m6A could be explained by the expression of complement regulators on the trophoblast membrane, which favors its activation. Regulatory proteins such as CD55 and CD59 are expressed on the trophoblast surface, protecting it against complement activity. CD55 degrades C3 and C5 convertase, while CD59 prevents MAC assembly (96). mRNAs with m6A tags in the CD55 and CD59 transcripts maintain their stability through IGF2BP; however, suppression of METTL3 could reduce CD55 and CD59 expression, promoting tissue damage in trophoblasts. This creates a feedback loop in which aPLs activate complement, releasing anaphylatoxins that can disrupt m6A regulatory enzymes, affect the expression of regulatory proteins and reducing complement inhibition, thus allowing for further amplification of the system, as reported in B-cell lymphoma (97, 98).

### 5.5 The aPL m6A tolerance axis: an integrative mechanistic model

The evidence discussed in sections 5.1 to 5.4 supports a model that places the m6A modification at the center of the non-thrombotic pathogenesis of OAPS. In this model, aPLs are not limited to the recognition of phospholipids in cell membranes. Rather, they affect post-transcriptional processes necessary to maintain HLA-G expression, immunological tolerance, Treg cell function, low cytotoxic activity of uNK cells, and complement control in the decidua (4).

It´s important to note that the sequence of events described below constitutes a proposed integrative hypothesis combining direct evidence from OAPS with findings extrapolated from other pathological and experimental models; this sequence has not yet been demonstrated as a unified mechanistic cascade in OAPS and should be interpreted as a framework to guide future experimental validation. First, circulating aPLs, particularly IgG1 and IgG3 with high affinity for domain I of β2GPI. These complexes bind to and interact with ApoER2 and TLR4, activating the p38 MAPK and NFκB pathways, increasing oxidative stress, and activating complement, which generates the first wave of m6A alteration through the destabilization of METTL14, the repression of METTL3 accompanied by the induction of ALKBH5, and the alteration of FTO activity. These events reduce the expression of key molecules in immune homeostasis such as HLA-G, PD-L1, CD200, galectin 1, CD55, and CD59 (21, 49).

During the amplification phase, the decrease in HLA-G promotes the activation of uNK cells and M1 macrophages, and the production of cytokines such as TNF-α, IL-1β, IL-6, and IFN-γ further suppresses METTL3, reinforcing the impaired function of trophoblasts, macrophages, NK cells, and Tregs. C5a amplifies this process via WTAP, increasing the dysfunction of these cells, while simultaneously reducing the expression of CD55 and CD59, which makes them vulnerable to complement damage (29). On the other hand, the reduction of FOXP3 mediated by m6A diminishes the anti-inflammatory profile (88).

The collapse phase of tolerance arises when interactions converge across multiple cellular compartments. Endothelial cells deficient in tolerogenic molecules become targets of uNK cells. This coordinated failure transforms the decidua into an inflammatory environment incompatible with trophoblast invasion, spiral artery remodeling, and fetal development (99). This model may help explain the heterogeneity of this pathology; fetal loss during the second trimester may be due to impaired EVT invasion, defective spiral artery remodeling, and NK cell-mediated trophoblast injury (100). Other late complications, such as preeclampsia and intrauterine growth restriction, may result from placental dysfunction driven by the m6A modification loop. Therefore, differences in aPL titers and m6A regulators such as METTL14, WTAP, FTO, ALKBH5, YTHDF2, and IGF2BP family members may determine the clinical phenotype (101).

This model generates several predictions. First, comprehensive transcriptomic analysis of placental m6A should be able to identify specific m6A signatures associated with altered transcripts of HLA G, FOXP3, KIR2DL4, CD55, and CD59. Second, restoration of m6A regulators should be able to restore trophoblast invasiveness, immune tolerance, and complement regulatory capacity. Third, the m6A methylation profile of decidual tissue resident cells could elucidate cell-type-specific signatures that correlate with the immune response and defects in placental establishment (70). Lastly, it could be a therapeutic target since its inhibition could simultaneously regulate several transcripts associated with tolerance. Taken together, this model provides an additional framework explaining how aPL-induced damage can trigger a series of events that favor the collapse of maternal-fetal tolerance (102) (**Figure 3**).

### 5.6 Context-dependent and divergent effects of METTL3, FTO, and ALKBH5

The mechanisms described in sections 3 through 5 explain how METTL3 and ALKBH5 participate in trophoblast invasion and immunological tolerance; however, these molecules act differently depending on the cell population, target transcripts, or stages of embryonic development. In the reviewed literature, m6A dysregulation attributed to the same enzyme can vary based on the biological context in which it was studied.

In the case of METTL3, loss-of-function models in human trophoblast stem cells and murine placentas demonstrate that depletion significantly reduces trophoblast invasion, spiral artery remodeling, and uteroplacental perfusion (45, 46, 71). However, this pattern is inconsistent with other observations: METTL3 expression and global m6A methylation have been reported to increase rather than decrease in the trophoblasts of patients experiencing preeclampsia, one of the clinical manifestations of OAPS. Furthermore, METTL3 behaves differently depending on the immune cell population. For example, in macrophages, METTL3 stabilizes anti-inflammatory transcripts such as IL-10 and SOCS1, promoting an M2 phenotype; conversely, in Treg cells, METTL3 stabilizes FOXP3 mRNA to maintain suppressive function a finding demonstrated in peripheral blood cells from patients with asthma (62, 67, 103). These findings indicate that METTL3 has transcript-specific functionality depending on the cell population; thus, one cannot assume that its dysregulation in the context of OAPS faithfully reflects findings from models of trophoblast invasion, specific cell subpopulations, or other obstetric pathologies.

ALKBH5 is another molecule that follows a similar pattern. Its overexpression reduces CYR61 mRNA stability and impairs trophoblast mobilization, which could be associated with impaired placental development (22) However, in murine models of hypoxia-associated pregnancy loss, it was demonstrated that the enzyme ALKBH5 exerts a protective effect against spontaneous abortion by demethylating SMAD1/5 (73). Since both effects are regulated by the same enzyme acting on different transcripts depending on the physiological context, ALKBH5’s role in OAPS cannot be inferred from any of the cited studies in isolation; this contribution likely depends heavily on the decidual microenvironment altered by aPLs.

FTO adds another layer of complexity, as it was initially characterized as a regulator of adipogenesis and energy metabolism before its activity as an RNA demethylase was discovered (51). Its proposed role as an HLA-G mRNA stabilizer has been explored in studies focusing on trophoblasts, and the association between FTO variants and recurrent miscarriage remains a mechanistic hypothesis lacking direct evidence regarding how aPL exposure modulates FTO expression and activity *in vivo* (82).

Finally, most of the analyzed evidence comes from first-trimester tissues, immortalized trophoblast cell lines, or murine models (45, 46) Given that METTL3 expression peaks in cytotrophoblasts during initial invasion and decreases as differentiation progresses, it is unknown whether METTL3, FTO, and ALKBH5 exert comparable effects in decidual cells at later stages of gestation (70, 74). Taken together, these studies argue against viewing the dysregulation of METTL3, FTO, and ALKBH5 as a single, unidirectional process in OAPS; instead, they support a model in which their contribution depends largely on the cell population, target transcripts, and gestational timing.

## 6 Therapeutic implications and future directions

### 6.1 Pharmacological targeting of the aPL–m6A–tolerance axis

Identifying m6A dysregulation as a potential factor contributing to the loss of immunological tolerance in OAPS could pave the way for the development of drugs addressing needs that anticoagulant therapy alone cannot meet. However, it is important to note that—unlike hydroxychloroquine and statins, for which experimental evidence exists from clinical trials and murine models of OAPS—the strategies discussed below are based on the general pharmacological potency or biochemistry of each agent; their specific effects on m6A regulators have not yet been evaluated in trophoblasts, decidual tissue, or OAPS models. Consequently, these proposals should be viewed as potential therapeutic alternatives that require validation in *in vitro* and *in vivo* models (104–106).

Among the approaches targeting the enzymes that remove m6A marks, selective ALKBH5 inhibitors—such as IOX1, ALK-04, and ALKBH5-IN-1—represent candidates of biochemical interest (**Figure 4**). These molecules counteract the upregulation of ALKBH5 driven by HIF-1α or NF-κB activation; these pathways can be triggered by immune complex recognition or the oxidative stress characteristic of OAPS. However, to date, these compounds have been used only as adjunctive therapies for acute myeloid leukemia and glioblastoma; none have yet been evaluated in trophoblast cell lines or OAPS, highlighting the need for further analysis of these molecules. Furthermore, an analysis of molecular subtypes in recurrent pregnancy loss of unknown etiology has linked the expression patterns of m6A regulators like ALKBH5 to the classification of the condition (22, 107). However, these studies have not been tested in hypoxia or aPL models to verify their effect on restoring trophoblast invasive capacity in OAPS.

**Figure 4:**
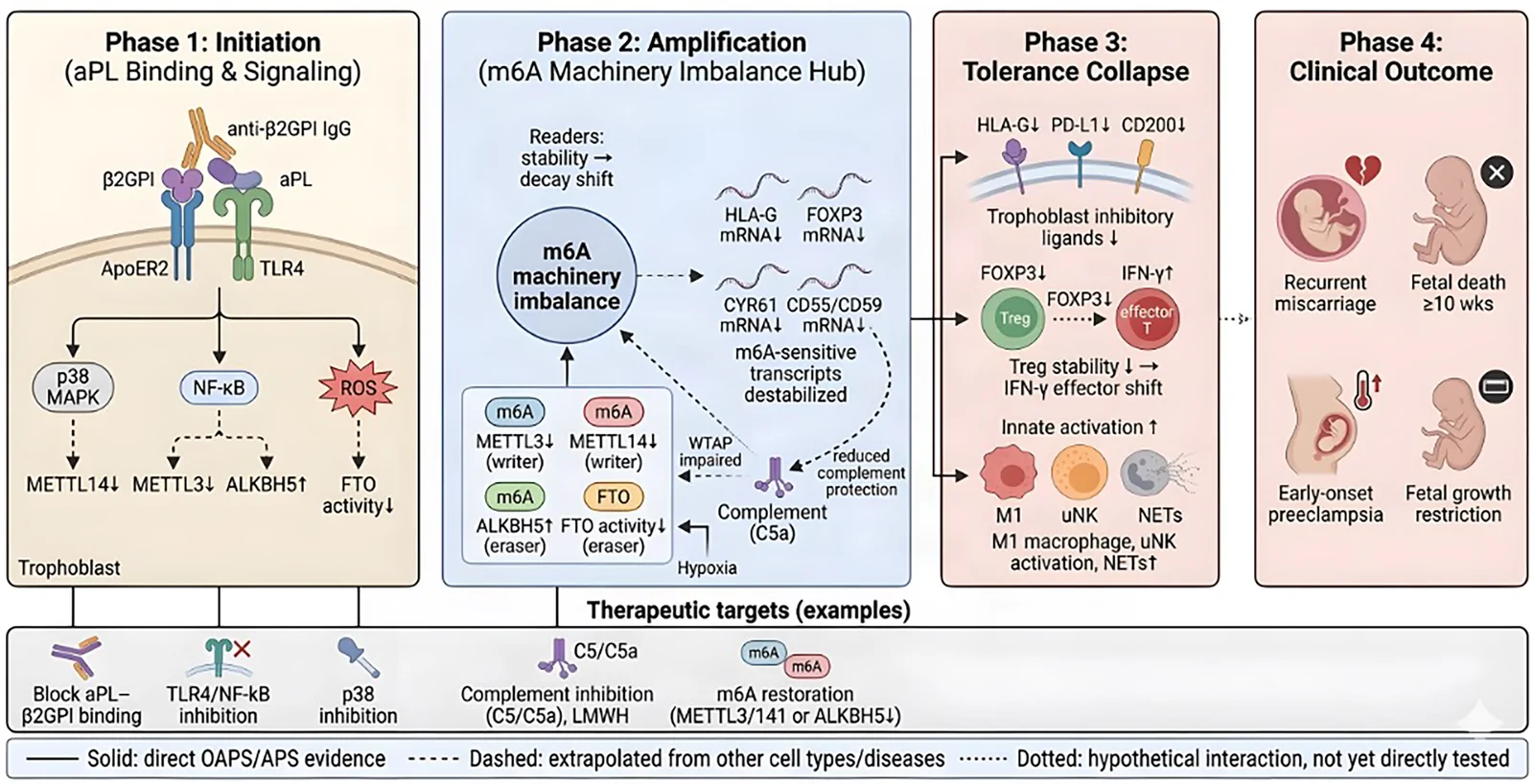
Pharmacological interventions targeting the aPL–m6A–tolerance axis in OAPS. Four-phase mechanistic model describing how aPLs dysregulate the m6A epitranscriptomic machinery and compromise maternal-fetal tolerance. (Phase 1) Anti-β2GPI binding to the trophoblast activates TLR4/ApoER2 and pathways such as p38 MAPK, NF-κB, and oxidative stress, inhibiting METTL3/METTL14/FTO and favoring ALKBH5. (Phase 2) Global alteration of m6A destabilizes key transcripts (HLA-G, CYR61, FOXP3, CD55/CD59), amplified by C5a signaling and hypoxic conditions. (Phase 3) Tolerance collapses with loss of immunoregulatory molecules, Treg dysfunction, uNK activation, M1 macrophage polarization, and increased NETs. (Phase 4) These alterations converge into placental dysfunction and vascular insufficiency, clinically manifesting as recurrent miscarriage, early preeclampsia, and fetal growth restriction.

Modulating the catalytic activity of m6A-regulating enzymes represents a potential complementary therapeutic approach. S-adenosylmethionine (SAM) can donate the methyl group required by m6A methyltransferases, whereas α-ketoglutarate acts as a crucial cofactor for the Fe2+-dependent demethylases FTO and ALKBH5; this could help restore the catalytic activity of these enzymes, which is impaired by mitochondrial dysfunction and oxidative stress induced by aPLs (108, 109).

Blocking YTHDF2 with antisense oligonucleotides could represent an additional strategy to promote mRNA stability for molecules involved in immunological tolerance—such as HLA-G, FOXP3, KIR2DL4, CD55, and CD59. These molecules are crucial for protection against complement and cytotoxic activity; their loss could lead to a breakdown of tolerance in the context of OAPS (68, 110, 111).

Among established therapies for the treatment of obstetric antiphospholipid syndrome (OAPS), hydroxychloroquine has demonstrated efficacy in preventing aPL-induced placental damage in murine models of the condition. Its mechanism of action involves inhibiting NF-κB activation in various cell populations, such as trophoblasts and macrophages; this makes it mechanistically plausible that the drug could influence METTL3 transcription via the NF-κB–HDAC axis. This relationship is of vital importance because, within the proposed aPL-m6A-tolerance axis, NF-κB signaling can influence the methylation pattern and the regulation of the proinflammatory microenvironment (71, 112).

Low molecular weight heparin (LMWH) may also exert effects beyond its classic anticoagulant activity. *In vitro* studies have shown that LMWH can influence trophoblast invasiveness and counteract aPL-mediated impairment of endometrial angiogenesis, while also affecting complement-regulated mechanisms during pregnancy. It should be noted, however, that effects on C5a-mediated WTAP phosphorylation or on CD55 and CD59 transcripts have not been demonstrated; thus, this remains a hypothesis (113–115).

Among the various candidates presented in this section, statins are supported by the most robust experimental evidence. Pravastatin has demonstrated benefits in improving pregnancy outcomes in patients with obstetric antiphospholipid syndrome (OAPS) refractory to conventional antithrombotic therapy. Its ability to suppress NF-κB activation has been shown in human peripheral blood monocytes, making it biologically plausible that it affects METTL3 expression; however, this has not been studied in the placenta or decidua, and a direct effect on ALKBH5 expression via HIF-1α destabilization remains speculative and unconfirmed (116–118).

Taken together, these observations suggest that pharmacological modulation could affect m6A enzyme complexes in patients with OAPS and yield beneficial effects. The conceptual framework presented is supported by indirect and largely extrapolated evidence; hydroxychloroquine and statins emerge as the strongest candidates for implementation alongside conventional treatment, although more robust clinical data are required for their adoption.

### 6.2 Limitations of the proposed model and future directions

Despite the evidence supporting the mechanistic framework proposed in this review, it is important to acknowledge that it has several limitations. In particular, evidence directly linking m6A modification dysregulation to the pathogenesis of OAPS remains scarce. To date, no studies systematically characterize the global m6A density, m6A profiles in the transcriptome, or the expression of m6A regulatory enzymes such as METTL3, METTL14, ALKBH5, and FTO in trophoblasts, decidual cells, placental tissue, or cells of the innate and adaptive immune systems (105). Therefore, the model presented in this review is based solely on the convergence of independent but biologically interconnected mechanisms. Among the molecular mechanisms that promote placental injury mediated by pluripotent stem cells (PSCs), m6A modification may emerge as playing a significant role in the development of gestational complications. Growing evidence supports the hypothesis that the m6A modification could contribute to the development of preeclampsia, intrauterine growth restriction, and recurrent miscarriages characteristic of post-occlusive disease (POD), by altering the immune response (22, 71, 72, 103, 107). However, although these observations support a possible contribution at the level of epitranscritomic regulation in the pathogenesis of POD, a causal relationship cannot yet be established.

To address these knowledge gaps requires various complementary lines of experimental evidence to validate the proposed model. First, a global epitranscriptomic profile of placental and decidual tissue must be generated. This involves m6A sequencing and MeRIP-seq analysis of tissues from patients with OAPS—classified according to the 2023 ACR/EULAR criteria and stratified by gestational age and clinical manifestations (e.g., early vs. late pregnancy loss, isolated fetal growth restriction, or superimposed preeclampsia) (119).

Second, analysis at the cellular level is essential. Given that placental tissue consists of a heterogeneous mixture of EVTs, decidual macrophages, Treg cells, and uNK cells, it is necessary to characterize the m6A methylation profile at the single-cell level or via MeRIP analysis of cell populations sorted by flow cytometry. This approach would allow for the identification of methylation patterns characteristic of each cell type—a factor that likely explains some of the discrepancies noted in Section 5.6 (22, 73, 103).

Third, specific validation of transcripts key to immunological tolerance, such as HLA-G and FOXP3, is required. Quantifying m6A density within DRACH motifs—using m6A-IP-qPCR or SELECT-qPCR—will enable correlation with HLA-G expression in EVTs and with the suppressive capacity of decidual Treg cells (assessed via functional suppression assays or co-cultures with effector T cells), comparing OAPS patients with gestational age-matched controls (29, 67, 88).

Fourth, functional gain- and loss-of-function studies targeting METTL3, FTO, and ALKBH5 are needed in physiologically relevant systems. Modulating these enzymes—via genetic or pharmacological strategies—in trophoblast organoids or EVT cell lines exposed to IgG or aPL from patients with OAPS, as well as in isolated decidual Treg cells, will allow for the direct assessment of functional parameters such as trophoblast invasiveness, apoptosis, FOXP3 expression, and uNK cell cytotoxic activity. Furthermore, validation in standardized *in vivo* OAPS models—such as the passive transfer of aPL is crucial to determine whether modulating m6A-regulatory enzymes (genetically or pharmacologically) is sufficient to restore maternal-fetal tolerance and improve pregnancy outcomes (45, 46, 53).

Finally, studies involving large-scale cohorts can clarify whether inter-individual variation in the m6A enzymatic machinery contributes to disease susceptibility. Analyzing polymorphisms and functional variants in genes encoding m6A-regulatory enzymes could help identify subgroups genetically predisposed to specific obstetric complications; this would strengthen causal inferences and guide the development of precision therapeutic approaches based on molecular mechanisms (8, 23, 106, 119, 120).

## 7 Conclusion

OAPS has traditionally been linked primarily to thrombotic mechanisms; however, evidence from recent years indicates that its pathogenesis involves a variety of immunological mechanisms that converge on disruption of the maternal-fetal interface, where epitranscriptomic mechanisms play a central role. The axis by which the presence of aPL induces changes in the m6A modification via the p38 MAPK, NFκB, and oxidative stress signaling pathways alters the functionality of trophoblasts and decidual immune cells. This results in reduced HLA-G expression, impaired trophoblast invasion, polarization of Tregs to effector T cells, increased uNK cytotoxicity, and decreased complement regulation, which together lead to fetal loss. This mechanism could explain the limited efficiency of current anticoagulant therapies, positioning m6A regulators such as METTL3, ALKBH5, FTO, and YTHDF2 as potential therapeutic targets, while establishing a basis for future validation through epitranscriptomic profiling and targeted intervention strategies.
